# Divergent Successional Patterns of *phoC*- and *phoD*-Phosphate-Solubilizing Microbes During Plateau Mammal (*Ochotona curzoniae*) Carcass Decomposition

**DOI:** 10.3390/microorganisms14010153

**Published:** 2026-01-09

**Authors:** Jie Bi, Xianxian Mu, Shunqin Shi, Xueqian Hu, Petr Heděnec, Maoping Li, Huan Li

**Affiliations:** 1 School of Public Health, Lanzhou University, Lanzhou 730000, China; bijie919@163.com (J.B.); mxx990517@163.com (X.M.); 15111708220@163.com (S.S.); huxueqian37va@163.com (X.H.); 2 Institute for Tropical Biodiversity and Sustainable Development, University Malaysia Terengganu, Kuala Terengganu 21030, Tganu, Malaysia; petr.hedenec@umt.edu.my; 3 State Key Laboratory of Herbage Improvement and Grassland Agro-Ecosystems, College of Pastoral Agriculture Science and Technology, Lanzhou University, Lanzhou 730000, China; 4 Center for Grassland Microbiome, Lanzhou University, Lanzhou 730000, China

**Keywords:** corpse decay, *phoC*-harboring microbes, *phoD*-harboring microbes, community assembly process, co-occurrence patterns

## Abstract

Microbial communities associated with animal cadaver decomposition play a crucial role in biogeochemical cycles in both aquatic and terrestrial ecosystems. However, it remains unclear regarding the diversity, succession, and assembly of phosphate-solubilizing microbes during animal cadaver decay. In this study, plateau pikas (*Ochotona curzoniae*) as mammal degradation models were placed on alpine meadow soils to study diversity, succession and assembly of phosphate-solubilizing microbes using amplicon sequencing of *phoC*- and *phoD*-genes during 94 days of incubation. The total phosphorus concentration in the corpse group increased by 8.53% on average. Alpha diversity of both *phoC*- and *phoD*-harboring microbes decreased in the experimental group compared to the control group, and the community structure differed between control and experimental groups. Phosphate-solubilizing microbial community turnover time rate (TDR) of the experimental group was higher than that of the control group, indicating corpse decay accelerates the succession of *phoC*- and *phoD*-harboring microbial community. Null model revealed that deterministic process dominated *phoC* microbial community in corpse group, while the stochastic process dominated *phoD* microbial community. The microbial network in experimental group was more complicated than that in control group of *phoC* microbial community, while *phoD* microbial community showed opposite trend. Partial least squares path modeling (PLS-PM) showed that *phoC*-harboring microbial community was mainly influenced by pH, Total carbon (TC) and Total phosphorus (TP), while the *phoD* microbial community was only regulated by TP. These findings elucidate the ecological mechanism of phosphorus-solubilizing microbial community changes during animal corpse degradation.

## 1. Introduction

Animal cadaver decay is a universal natural phenomenon [1]. As wild animal bodies decompose, various cadaveric components such as fat, carbohydrates, and proteins are released into the soils, and thus impact the biogeochemical cycles of carbon, nitrogen, phosphorus, and sulfur [2]. Study has shown that a corpse typically contains about 20% carbon, which serves as an important source of available nutrients [3]. Cadaver decomposition is also an important source of other nutrients such as nitrogen and phosphorus. For example, a study by Towne [4] found that the concentration of nitrogen and phosphorus within 50 cm of the decomposing cadavers is significantly higher than that of the surrounding soils. These local nutrient pulses make a concentrated fertility island around the corpse soils, which is also called corpse decomposition island (CDI) [3]. The CDI is a microecosystem that contains microorganisms such as bacteria and fungi, which can change the biodiversity of the surrounding environment [5]. Previous studies have shown that decomposition of cadavers increases the network complexity and time turnover rate of carbon-fixing and nitrogen-circulating microorganisms, while there is little research on P-cycling microbes [6,7].

Phosphorus (P) is an essential nutrient for energy metabolism and cellular structure of all organisms [8]. Since P is not available in the atmosphere, it is considered the second most limiting nutrient after nitrogen [9]. Approximately 43% of land is predominantly constrained by phosphorus, which is prone to being easily lost from the soils [10,11]. It is crucial to understand the dynamics of the phosphorus cycle during the corpse decomposition which in turn affects nutrient utilization [12]. The increase in total phosphorus levels in soils does not necessarily link with the increase in available phosphorus for plants [13]. For example, organic phosphorus (Po) released to soils during corpse decomposition is difficult to be directly absorbed by plants [14]. The Po is converted into accessible inorganic phosphorus (Pi) via microbial activity of bacteria in soils [15]. The transformation of Po into Pi primarily occurs through three mechanisms: (i) participation in inorganic phosphorus solubilization and organic phosphorus mineralization; (ii) phosphorus absorption and transport; (iii) and regulation of phosphorus starvation reactions [16,17]. Mineralization of Po is an important step to control phosphorus cycle in soils. Soil microbes are involved in the mineralization of P via producing extracellular enzymes, in particular by phosphatase enzymes [18] and resulting in the release of bioavailable orthophosphate [19]. Acidic (ACP, EC 3.1.3.2) and alkaline (ALP, EC 3.1.3.1) phosphatase activities serve as markers for assessing the conversion of organic phosphorus into bioavailable inorganic phosphate [20]. The *phoC* gene is a microbial functional gene encoding non-specific acid phosphatase, while *phoD* is a functional gene encoding alkaline phosphatase activity [20]. These functional genes are key members of the phosphorus cycling genes (PCG) involved in organophosphorus mineralization [17]., Most bacteria harbor these phosphate (*Pho*) regulators, categorizing them as phosphorus cycling microorganisms (PCMs) [21,22]. Studying the community dynamics of PCMs in cadaver decomposition succession can offer insights into microbial genes, thereby enhancing our comprehension of the phosphorus cycle in natural environments.

Understanding the changes in microbial community composition and structure associated with nutrient transformation has become a hot topic in recent microbial ecology [23]. A crucial aspect in ecology is comprehending the temporal dynamics of microorganisms to gain insights into ecosystem processes. Time–decay relationships are employed to elucidate the variations in the similarity of intricate and diverse microbial communities over time [24]. Time turnover rate (TDR) is defined as the number of species that are eliminated and replaced by classification operation units over time [25]. Given that changes in the composition of microbial communities are often associated with changes in their functional capacity, understanding the temporal dynamics of microbes is crucial for understanding ecosystem processes [26]. Studies have shown that stochastic processes and deterministic processes are two processes that govern the aggregation of species into communities [27,28,29]. The examination of the relative impacts of deterministic processes (biological interactions and environmental filtering) and stochastic processes (random birth, diffusion, and drift) on community assembly is a key focus in microbial ecology [30]. Furthermore, the microbiome typically operates within intricate species interaction networks rather than in isolation, which to some extent regulates the structure of microbial populations [31]. The terms “competition” and “cooperation” are utilized to describe interactions within communities that contend for limited resources [32]. Synthesizing these methods to study microbial community dynamics will help us to enhance our understanding of how corpse decomposition influences the community succession of *phoC* and *phoD* PCMs.

The Qinghai–Tibet Plateau is a climate-sensitive alpine meadow ecosystem and a critical indicator of global change, with its responses to disturbance foreshadowing trends in similar ecosystems worldwide [33,34,35]. In this nutrient-limited environment, nitrogen (N) and phosphorus (P) are key restrictive factors for plant productivity, with their dynamics tightly coupled [36,37]. Therefore, studying microorganisms in the nutrient-poor soils of the Qinghai–Tibet Plateau not only provides insights into local ecological issues but also helps us understand how cold, nutrient-poor ecosystems worldwide respond to external disturbances. We choose *Ochotona curzoniae* as the decomposition model of animal carcasses because it is a local species widely existing in the Qinghai–Tibet Plateau and has a high population density and the mortality rate is high [38,39], thus its corpse is suitable for study as a model. In this study, the influence of wild animal carcasses decay on the PCMs in alpine meadow soils of Qinghai–Tibet Plateau was studied by sequencing *phoC* and *phoD* functional genes, with pikas as model animal. Building upon prior research, a scientific hypothesis was proposed: the carcass decomposition process enhanced soil nutrients and altered soil physicochemical properties [2], resulting in diverse shifts in *phoC*- and *phoD*-PCMs community composition, assembly mechanisms, and co-occurrence networks of PCMs carrying *phoC* and *phoD* genes [40,41]. The validity of the hypothesis was evaluated by addressing the following questions: (1) Does corpse decomposition affect the composition and diversity of *phoC*- and *phoD*-PCMs? (2) How does corpse decomposition influence the community assembly mechanisms of *phoC*- and *phoD*-PCMs? (3) What is the effect of corpse decay on the co-occurrence patterns of bacterial networks *phoC*- and *phoD*-PCMs? (4) What is the potential pathway through which the decomposition process affects the microbial communities of *phoC* and *phoD* genes by altering physicochemical factors? The findings of this study revealed the response mechanism of phosphorus-solubilizing microbial communities to the stress of animal carcass decomposition, providing a theoretical foundation for evaluating microbial functions in ecosystems globally following disturbance.

## 2. Materials and Methods

### 2.1. Experimental Procedures and Sampling

From June to September 2017, we conducted a 94-day outdoor experiment. Soil samples from the surface layer (0–10 cm) were collected using shovel from the Qinghai–Tibet Plateau in Haibei Tibetan Autonomous Prefecture (37°31′ N, 101°19′ E). Subsequently, the soils were sieved with a 2 mm mesh size to remove stones and plant debris and placed into fifty pots (d = 30 cm, h = 20 cm) so that each pot contained 3 kg of soils. Additionally, twenty-five plateau pikas were captured from the local habitat, euthanized using ether, and randomly positioned on the surface of twenty-five pots to serve as the experimental group (EG). The remaining twenty-five flowerpots were designated as the control group (CG) to replicate the decomposition conditions of the carcass. The experiment was conducted outdoors on the Qinghai–Tibet Plateau, where temperatures ranged between 12 and 18 °C on average. Destructive sampling was performed at five time points (Day 4, 7, 12, 20, and 94). At each time point, five pots were randomly selected from each treatment group (EG and CG). Soil samples (50 g each time) from the depth of 0–10 cm beneath. Each pot was sampled only once. This sampling scheme resulted in a total of 50 independent soil samples (2 treatments × 5 time points × 5 biological replicates per combination). Soil samples were stored at −20 °C for downstream analyses.

### 2.2. Detection of Soil Physicochemical Properties

The physicochemical characteristics of soils, such as pH, total carbon (TC), total phosphorus (TP), and total nitrogen including ammonium nitrogen (NH_4_-N) and nitrate nitrogen (NO_3_-N), were examined in our study [7,42]. Specifically, pH levels were measured using a desktop pH meter (AZ86505, Shenzhen Lemaiyi Electronics Co., Ltd., Shenzhen, China), while TC TP, and TN were analyzed using a total organic carbon analyzer (Shimadzu Corporation, Kyoto, Japan), ultraviolet colorimeter (Thermoscientific, Waltham, IL, USA), and Kjeldahl nitrogen analyzer (Shanghai Hongji Instrument Co., Ltd., Shanghai, China), respectively. The determination of NH_4_-N and NO_3_-N was carried out using the Indophenol blue (IPB) method and an ultraviolet colorimeter, respectively. The changes in soil physical and chemical properties with time in experimental group (EG) and control group (CG) are shown in the annex (Figure S2, Tables S1–S3).

### 2.3. DNA Extraction, Amplicon Sequencing and Bioinformatics Analysis

The DNA was extracted from 0.2 g of frozen soil samples using the Soil Ezup Genomic DNA extraction kit from Sangon Biotechnology, Shanghai, China. The DNA concentration was quantified by Nanodrop 2000 spectrophotometer (Thermoscientific, Waltham, IL, USA) and diluted up to 10 ng/μL for further metabarcoding PCR amplification. Primer F (CGGCTCCTATCCGTCCGG) and Primer R (CAACATCGCTTTGCCAGTG) were used for PCR amplification of *phoC* gene [43] and Primer F (CAGTGGGACGACCACGAGGT) and Primer R (GAGGCCGATCGGCATGTCG) were used for amplification of *phoD* gene [44]. The detailed PCR protocol is provided in the Supplementary Materials. The amplified products were separated, purified, and recovered by 1% agarose gel electrophoresis, and then equimolarly mixed in Illumina MiSeq sequencer (Illumina, San Diego, CA, USA) for sequencing and analysis. Then, the original sequences are analyzed by bioinformatics using quantitative insights into microbial ecology (QIIME) pipeline, and the detailed information generation analysis steps refer to our previous study [7]. To sum up, using FLASH2 software (version 2.2.00) [45], Fastxtoolkit and Usearch 8.0 to eliminate low-quality sequences, high-quality and high-reliability clean reads can be obtained [46]. Then the remaining sequences were grouped into operational taxonomic units (OTU) with 97% nucleic acid similarity by using UCLUST algorithm [47]. Using blastp, the representative sequences of *phoC* and *phoD* were labeled based on the functional gene database of FunGene, and two final OTU tables were obtained [48]. Next, “daisy-chopper” script is used to obtain the same number of sequences per sample, so as to reduce the influence of uneven sequencing [49]. At the OTU level, we obtain sparse curves to evaluate whether the sequencing depth is sufficient (Figure S1). The observed OTU value (Alpha diversity) was calculated in the QIIME pipeline, and the beta diversity and community similarity between groups were calculated by the Bray–Curtis distance matrix: Community similarity = 1 − community dissimilarity [50]. Some microbes whose genera were not identified were called unclassified genera (UG), such as Gaitheraceae (UG) and Bacteroides (UG).

### 2.4. Time Decay Relation (TDR)

Time decay relation (TDR) was used to evaluate the change in community similarity with succession time, and a linear model was fitted based on the logarithm of community similarity and time of Bray–Curtis distance matrix. The TDRs calculation method involves the equation: log10(S) = constant − w log10(T), where t represents time interval, S denotes the pairwise similarity between samples, slope w represents the rate of community change, and the constant represents the intercept of the TDRs equation on a logarithmic scale [23].

### 2.5. Analyses of Community Assembly Process

We used two models commonly common in ecology to study the community assembly of bacteria harboring *phoC* and *phoD* genes. Based on the NCM, “Hmisc” software package (version 5.1-1) in R v 4.3.2 was used to calculate the influence of migration rateon the construction of *phoC*- and *phoD*-PCMs [51]. In this model, the parameter m represents the migration rate, with higher values indicating faster migration speeds of microbial communities and reduced diffusion constraints. The R-squared (R^2^) value reflects parameter fitting through nonlinear least squares fitting. A higher R^2^ value signifies better fitting, suggesting stochastic processes are more important. Based on the null model, the dominant role of stochastic and deterministic processes in the assembly of *phoC*- and *phoD*-PCM was calculated by using the “NST” software (version 3.0) package through R v 4.3.2 and evaluated by the modified random ratio (MST) value. With an MST value of 0–0.5 indicating deterministic dominance and a value of 0.5–1 indicating stochastic dominance [52]. Then, the important environmental variables driving microbial community assembly (MST value) were detected through the “random forest” package (version 4.7-1.1) of R v 4.3.2 [53]. The average increase in mean square error (%IncMSE) reflects the rise in mean square error upon variable removal, indicating the relative importance of the variable. Select significant environmental variables through the Permutation test.

### 2.6. Co-Occurrence Network

We constructed a network for the experimental group (EG) and the control group (CG) of *phoC* and *phoD* microbial community, respectively, to explore the influence of corpse decay on the co-occurrence pattern between phosphorus-solubilizing microbial communities. We selected the top 200 OTUs (average relative abundance > 0.1%) as the dominant OTUs [54]. And we calculated their spearman rank correlation through the “psych” package (version 2.3.6) in R v.4.3.2, with only the correlation coefficient of Spearman |*r*| > 0.6 and OTU with *p* < 0.05. Visualization is carried out in Gephi v.0.9.2 [55]. It is evaluated by calculating six parameters (average degree, average path length, modularity degree, diameter, density, and average clustering coefficient) of network topology characteristics. Higher average degree, graph density, and clustering coefficient values mean higher network connectivity, while lower average path length and network diameter indicate closer network connectivity [56].

### 2.7. Partial Least Squares Path Modeling

Partial least squares path modeling (PLS-PM) was used to analyze the linear statistical relationship between multiple groups of variables [57]. The variables included in PLS-PM model include corpse, soil pH, NO_3_-N, NH_4_-N, pH, TC, TN, TP, and bacterial community composition represented by PCoA1 and PCoA2 [58]. The model is constructed by using “plspm” (version 0.5.0) in R v.4.3.2. Goodness-of-fit (GoF) is a value to evaluate the explanatory degree of the model. R^2^ is the explanatory degree of variance [59].

### 2.8. Statistical Analysis

Kruskal–Wallis test and pairwise comparisons were used to compare the MST and the differences in soil physical and chemical properties between the experimental group and the control group at five time points in SPSS 26.0 (IBM Inc., Armonk, NY, USA) [60]. Then two-way analysis of variance (two-way ANOVA) was used to compare the effects of corpse addition and succession time on soil physical and chemical properties [61]. *t*-test was used to compare the differences in *phoC* and *phoD* phosphate-solubilizing microbial communities. Prior to ANOVA and *t*-test, the assumptions of normality (Shapiro–Wilk test on residuals) and homogeneity of variances (Levene’s test) were verified. Mann–Whitney *U* test was used to compare the differences in observed OTUs between the experimental group and the control group at each time point. The principal coordinate analysis (PCoA) matrix based on Bray–Curtis distance was used to analyze the differences in community structure between *phoC* and *phoD* phosphate-solubilizing microbes, and the differences in beta diversity and environmental factors were tested by the “vegan” software (version 2.6-6) package in R v 4.3.2 with permutational multivariate analysis of variance (PERMANOVA) [62]. Venn diagram of OTUs shared by EG group and CG group of *phoC* and *phoD* harboring microbes was drawn through http://www.ehbio.com/test/venn/#/ (accessed on 21 March 2024) online website [63]. We also use the packages of “psych” (version 2.3.6) and “reshape2” (version 1.4.4) and “pheatmap” (version 1.0.12) of R v 4.3.2 to draw the correlation heatmap between network topology and physical and chemical factors [64]. The rest of the graphics are drawn by Origin 2021 (Origin Lab, Northampton, MA, USA).

## 3. Results

### 3.1. Effects of Cadaver Decomposition on phoD and phoC Harboring Microbial Populations

**Figure 1:**
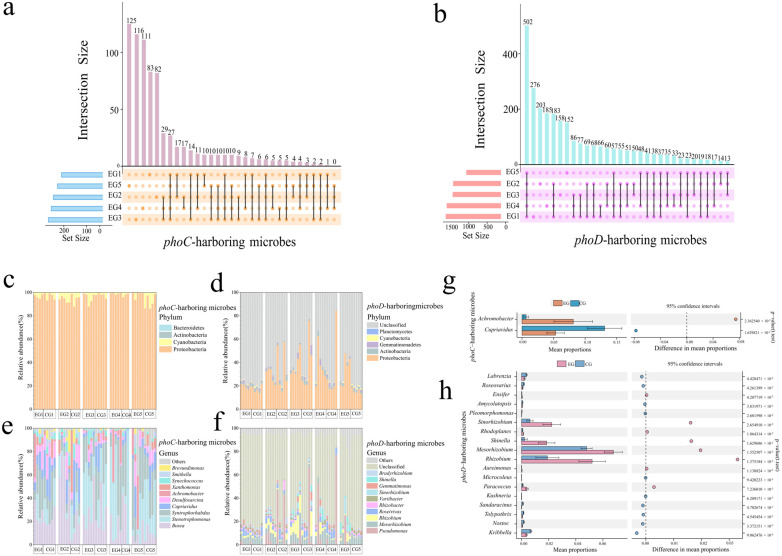
Differences in genera and shared OTUs between the EG and CG group of phoC and phoD harboring microbies during corpse decay. The diagram shows the shared OTUs in phoC harboring microbies (**a**) and phoD harboring microbies (**b**) across time points within the EG group. The percentage accumulation histogram shows the composition of phylum level in phoC (**c**,**d**) and phoD (**e**,**f**) harboring microbes in the EG and CG groups. The histogram shows the differential genera between the EG group and CG group in phoC harboring microbies (**g**) and phoD harboring microbies (**h**). Abbreviations: EG, experimental group; CG, control group.

The Venn diagram showed that among all the samples of *phoC* functional microbes, 363 OTUs were endemic in the experimental group (EG), accounting for 31.59%, while 400 OTUs were endemic in the control group (CG), accounting for 34.81%. Among all samples of *phoD* microbes, 539 OTUs were endemic to experimental group (EG), accounting for 15.71%, and 755 OTUs were endemic to the control group (CG), accounting for 22% (Figure S3). During cadaver decomposition succession, the EG group of *phoC*-harboring functional microbes shared 27 OTUs; the EG group of *phoD*-carrying functional microbes shared 502 OTUs (Figure 1a,b). At the phylum level, *phoC*-carrying microbes belonged mainly to Ascomycetes, followed by Cyanobacteria. As for the *phoD*-carrying microbes, 66.54% of the microbes were unclassified, followed by Ascomycetes, Actinobacteria, Tanystroemia and Cyanobacteria (Figure 1c,d). The dominant genera of *phoC*-microbes were *Bosea*, *Stenotrophomonas*, *Syntrophorhabdus*, *Cupriavidus* and *Desulfosarcina*, the average abundance ratio of unclassified microbes at the genus level carring *phoD* genes was 64.63%, followed by *Pseudomonas*, *Mesorhizobium*, *Rhizobium* and *Roseivivax* (Figure 1e,f). *Achromobacter* was more abundant in the EG group at the level of *phoC* phosphate-solubilizing microbes (*p* < 0.05), the abundance of *Mesorhizobium*, *Rhizobium*, *Sinorhizobium*, *Shinella*, *Paracoccus*, *Ensifer* and *Aureimonas* was higher in EG group of *phoD* microbes (Figure 1g,h). In conclusion, the composition of *phoC* and *phoD* phosphate-solubilizing microbes varied throughout the stages of corpse decomposition.

### 3.2. The Influence of Succession of Cadaver Decay on the Community Structure of phoC and phoD Phosphate-Solubilizing Microbes

**Figure 2:**
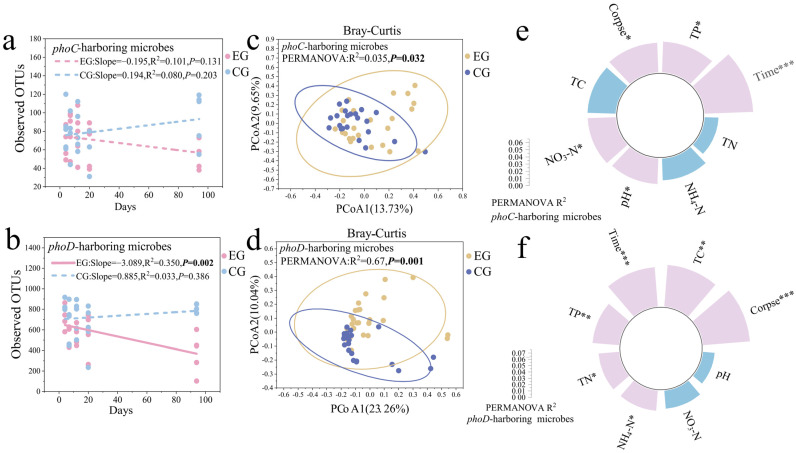
Differences in diversity of microbial community carrying phoC and phoD between experimental group and control group. Alpha diersity of phoC (**a**) and phoD (**b**) harboring microbes in EG and CG group. Principal coordinates analysis (PCoA) shows the beta diversity between EG and CG group of phoC (**c**) and phoD (**d**) microbes based on Bray–Curtis distance. The yellow and blue circles in (**c**,**d**) represent the confidence intervals for the EG group and CG group, respectively. The confidence level for this study is 0.95. The ring diagram shows the influencing factors that lead to the difference between EG group and CG group in phoC (**e**) and phoD (**f**) microbial community structure. Abbreviations: EG, experimental group; CG, control group. *: *p* < 0.05; **: *p* < 0.01; ***: *p* < 0.001.

We used the observed OTUs index to represent the alpha diversity of microbes. The observed OTUs of *phoC* functional microbes did not change much in the EG group and the CG group at five time points, and the alpha diversity of *phoD* functional microbes was significantly higher in the CG group than in EG group on the 94th day of succession (Figure S4a,b). Linear fitting showed that the alpha diversity of *phoC* functional microbes in the EG group decreased with the succession (Figure 2a, Slope = −0.195, R^2^ = 0.101, *p* = 0.131), while that in the EG group increased (Figure 2a, Slope = 0.194, R^2^ = 0.080, *p* = 0.203), as for *phoD* phosphate-solubilizing microbes, the observed OTUs of EG group decreased significantly compared with the control group (Figure 2b, Slope = −3.089, R^2^ = 0.350, *p* = 0.002). Therefore, the decomposition of corpse significantly reduced the alpha diversity of *phoD* microbial community. The decomposition of corpse had a significant effect on the community structure of *phoC* and *phoD* phosphate-solubilizing microbes. PCoA showed that both *phoC* and *phoD* microbes were significantly aggregated in EG group and CG group respectively (Figure 2c,d). Considering the variation in beta diversity of *phoC* and *phoD* microbes over succession time, there were significant differences between them in EG5 and CG5 groups (i.e., day 94) (Figure S4c,d).

**Figure 3:**
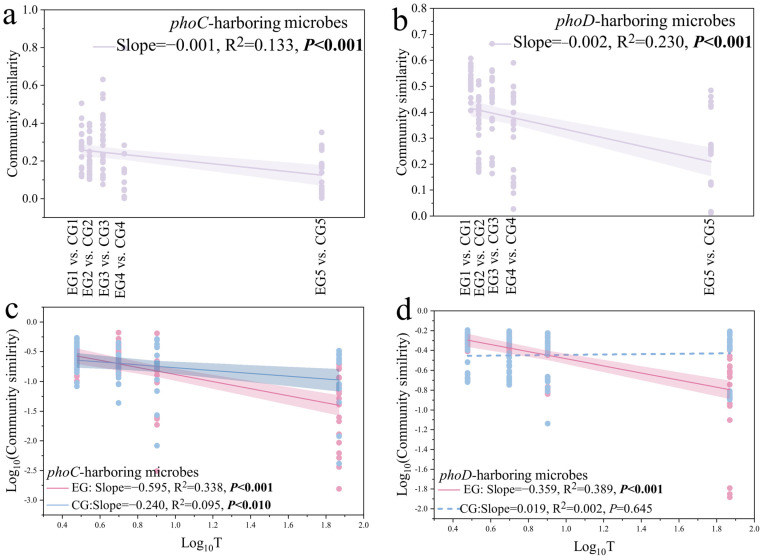
Community similarity (**a**,**b**) and temporal succession rate (TDR) (**c**,**d**) of phoC and phoD microbial communities.

PERMANOVA showed that the influencing factors of *phoC* harboring microbial community structure was cadavers, succession time, NO3-N, TP and pH, while *phoD* harboring microbial community structure was influenced by cadavers, succession time, NH4-N, TP, TC, and TN (Figure 2e,f). During cadaver decomposition, the community similarity between EG group and CG group of microbes carrying *phoC* and *phoD* genes decreased significantly (Figure 3a,b). The time decay relation (TDR) results showed that, compared to the CG group, corpses accelerated the time turnover rate of microbial communities carrying *phoC* and *phoD* in the EG group (Figure 3c,d).

### 3.3. Effect of Cadaver Decomposition on Community Assembly Mechanism of phoC and phoD Associated Microbial Communities

**Figure 4:**
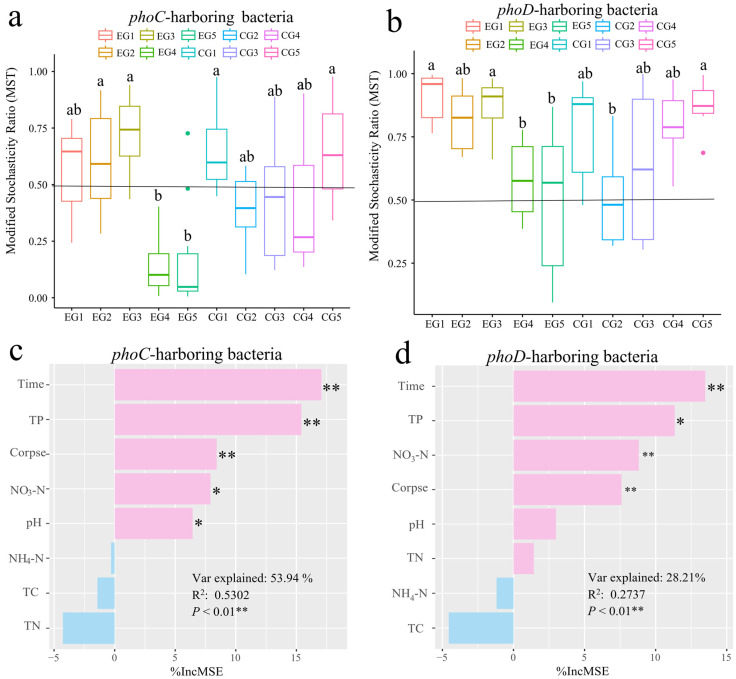
Community assembly mechanism of phoC and phoD microbes and physical and chemical factors affecting community assembly (**a**,**b**) The modified stochasticity ratio (MST) based on null modle of phoC and phoD communities in all samples. (**c**,**d**) The environmental variables in driving phoC and phoD microbies community assembly by random forest (RF). The value of %IncMSE (increased in mean squared error) means the importance of variables. *: *p* < 0.05; **: *p* < 0.01.

According to the result of MST, the CG group of functional microbes carrying *phoC* gene changed from the stochastic processes to the deterministic process on the 7th day (CG2), but returned to the stochastic process leading on the 94th day (Figure 4a, MST_CG1,5_ > 0.5, MST_CG2,3,4_ < 0.5), while the EG group of *phoC*-PCMs changed from the stochastic process to the deterministic process (Figure 4a, MST_EG1,2,3_ > 0.5, MST_EG4,5_ < 0.5). Whether in the EG group or the CG group, the process of community assembly of microbes carrying *phoD* gene was dominated by stochastic process (Figure 4b, MST_EG_ = 0.739, MST_CG_ = 0.716). We also fitted *phoC* and *phoD* microbial communities into neutral community models (NCM). The results showed that the migration rate of microbes carrying *phoC* gene in the EG group was 0.002, that of the CG group was 0.004, the m value of microbes carrying *phoD* gene in the EG group was 0.199, and that of the CG group was 0.491. The addition of animal carcasses reduced the mobility of microbes carrying *phoC* and *phoD* in the soils of the Qinghai–Tibet Plateau (Figure S5). Furthermore, the contribution of physical and chemical factors to MST value was calculated according to random forest analysis to explore the influencing factors of *phoC* and *phoD* microbes community construction. Results showed that contribution of physiochemical properties to the aggregation of microbial community carrying *phoC* gene and *phoD* gene were different. Time, TP, corpse, NO_3_-N, and pH were the important driving factors for the microbial communities carrying *phoC* gene, while the construction process of microbial community carrying *phoC* gene was significantly influenced by time, TP, NO_3_-N and corpse (Figure 4c,d).

### 3.4. Effect of Corpse Decomposition on Network Co-Occurrence Pattern of Microbes Carrying phoC and phoD Genes

**Figure 5:**
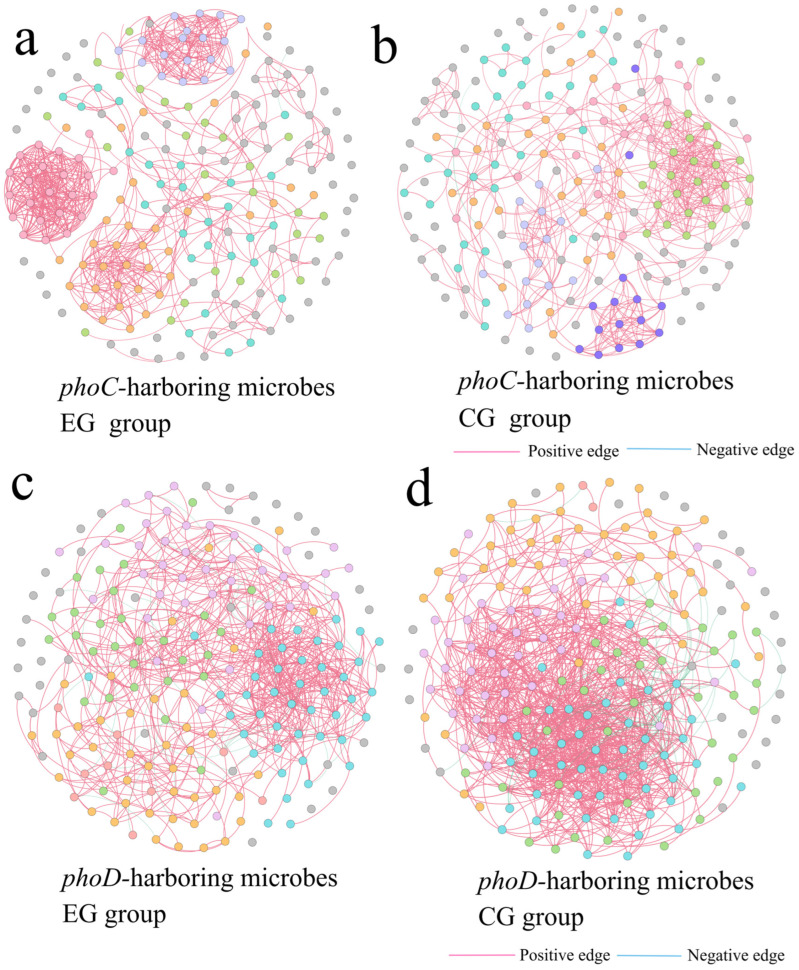
Co-occurrence network of EG and CG groups in *phoC* (**a**,**b**) and *phoD* (**c**,**d**) microbial communities.

**Figure 6:**
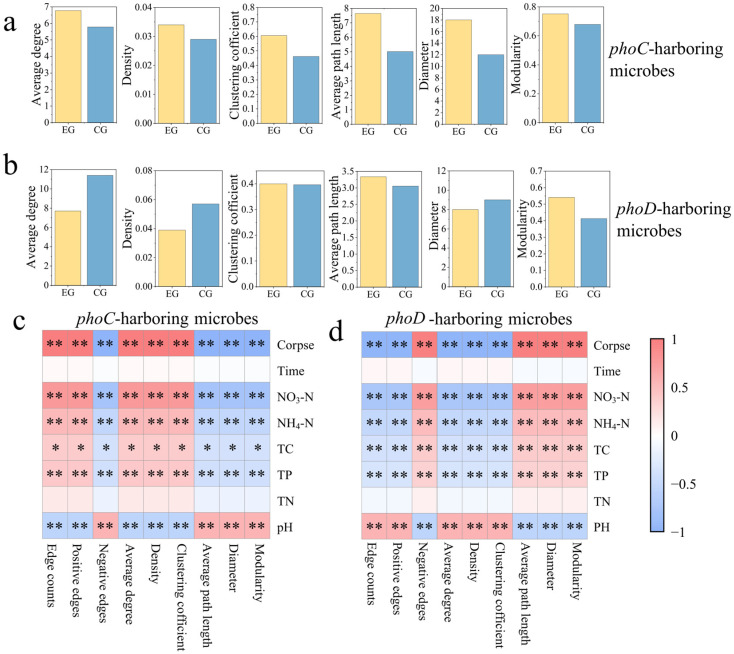
Topology characteristics (**a**,**b**) and their Spearman’s correlations with physical and chemical factors of soil (**c**,**d**) in *phoC* and *phoD* microbial communities. *: *p* < 0.05; **: *p* < 0.01.

By constructing a symbiotic network, the potential influence of adding corpses on microbes carrying *phoC* and *phoD* networks was evaluated. OTUs with the top 200 relative abundance were included in the network. Specifically, the EG group of *phoC* microbial network consisted of 65 points and 30 edges, the CG group consisted of 66 points and 26 edges (Figure 5a,b). The EG group of *phoD* microbial network consisted of 162 points and 575 edges, and the CG group consisted of 163 points and 850 edges (Figure 5c,d). This suggested that *phoD* microbial network was more complex than *phoC* microbial network. Network topological features were shown by Figure 6a,b. The number of edges, average degree and graph density of the EG group of the microbial network carrying the *phoC* gene were higher than those of the CG group, but the opposite was true for the network of the microbial community carrying the *phoD* gene.

We further calculated the Spearman correlation between network topology characteristics (edge counts, positive edge rate, negative edge rate, average degree, density, clustering coefficient, average clustering length, network diameter and modularity) and influencing factors to determine the different influencing factors of *phoC* and *phoD* networks (Figure 6c,d). Topological characteristics of microbes carrying *phoC* gene, such as positive edge rate, average degree, graph density, average path length and network diameter, were positively correlated with corpse addition, NO_3_-N, NH_4_-N, TC and TP, and negatively correlated with pH, while the situation of microbes carrying *phoD* gene was opposite.

### 3.5. Path Model of the Influence of Corpse Decomposition on phoC and phoD Phosphate-Solubilizing Microbes

**Figure 7:**
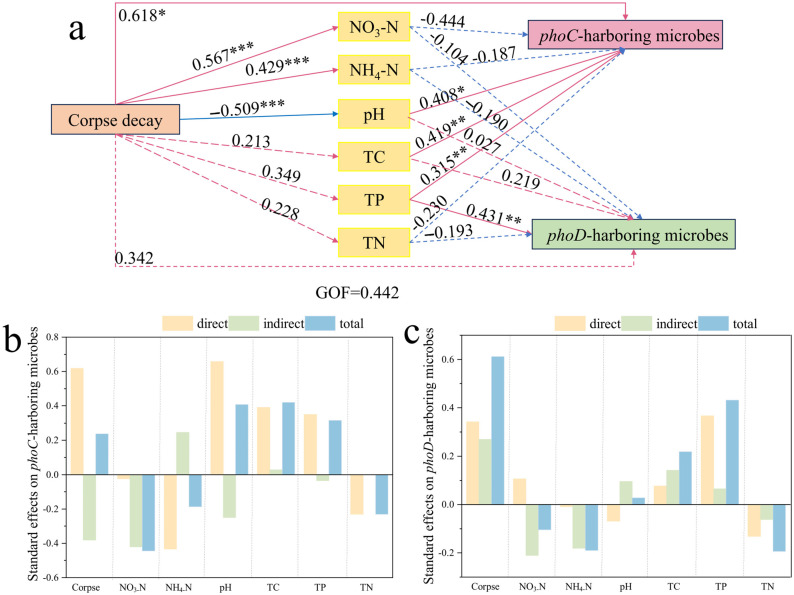
(**a**) Partial least squares path modeling (PLS-PM) showing significant direct and indirect effects of corpse, soil physicochemical factors on *phoC* and *phoD* harboring microbial communities. Solid and dashed arrows denote positive and negative relationships, respectively. R2 denotes the proportion of variance explained. The histogram shows the standardized effect of influencing factors on *phoC* and *phoD* microbies (**b**,**c**). *: *p* < 0.05; **: *p* < 0.01; ***: *p* < 0.001.

PLS-PM revealed the possible way that the decomposition of cadavers regulated soil *phoC* and *phoD* functional microbes through the influence on physicochemical properties. The results showed that the decomposition of corpse significantly affected *phoC* microbes in two ways. First, the adjustment of pH affected *phoC* microbes. Second, it directly affected *phoC* functional microbes. However, with the prolongation of cadaver decomposition succession, the concentration of TP increased, thus regulating the abundance of *phoD* microbes (Figure 7a). According to the standardization effect of PLS-PM, soil *phoC* microbes were mainly affected by pH, and soil *phoD* microbes were mainly affected by TP (Figure 7b,c). In summary, cadaver decay changed the physicochemical properties of soils and regulated the phosphorus-melting microbes community.

## 4. Discussion

### 4.1. Alterations in Soil Microbes Containing phoC and phoD Genes During Plateau Pikas Cadaver Decomposition

The percentage stacking chart combined with histogram showed that some phosphorus-solubilizing microorganisms increase significantly, while others decrease significantly (Figure 1). The process of cadaver decomposition exerts a discernible impact on various microbial communities, leading to alterations in the composition and prevalence of *phoC* and *phoD* microbes within the experimental groups. Our results revealed that *Proteobacteria* was the main carrier of *phoC* and *phoD* genes. In addition, there was a significant increase in the abundance of *Achromobacter*, a key microbial genus housing the *phoC* gene, which contains cells equipped with the C-P lyase system known to facilitate the solubilization of organic phosphate [65]. Moreover, microbes carrying the *phoD* gene were also found to enhance phosphorus release, exemplified by the increased prevalence of *Mesorhizobium*, *Rhizobium*, and other species in the experimental setting. In agreement with Lang et al., we suggest that *Mesorhizobium* is the main predictor of the concentration of orthophosphate monoester (OM) and organophosphate (Po), and also the main predictor of ALP activity [66]. *Rhizobium* can form root nodules of leguminous plants, thus symbiotic with leguminous plants, providing available inorganic nitrogen and inorganic phosphorus (Pi) for plants [67,68]. Phosphorus-solubilizing microorganisms (such as *Burkholderia* sp. and *Penicillium* sp.) release organic acids and enzymes, which significantly improve the availability of soil phosphorus, reduce the application amount of phosphate fertilizer, and increase rice yield [69]. Growth-promoting bacteria can improve the availability of phosphorus in plants by dissolving soil fixed phosphorus and secreting acid phosphatase, thus promoting plant growth and yield [70]. In addition, studies have shown that the fixation of phosphorus by microorganisms will also affect the carbon sequestration potential and alpine meadow productivity [71].

### 4.2. Decrease in Microbial Community Diversity and Community Similarity of Phosphorus Cycle Function Genes During Plateau Pikas Corpse Decay

The process of corpse decomposition leads to differences in community diversity of phosphate-solubilizing microbes. Compared with the control group, the observed OTUs of phosphorus-solubilizing microbes carrying *phoC* and *phoD* genes decreased, indicating that composition of phosphorus-solubilizing microorganisms gradually simplified under the decomposition pressure of carcass. Previous research has demonstrated carcass decomposition reduces the alpha diversity of carbon sequestering and denitrifying microbial communities, highlighting the significance of decomposition in biogeochemical cycling [7,72]. In addition, the structures of phoC or phoD harboring microbes between EG and CG groups were significantly separated, the community similarity decreased. In this study, TP in the EG group was significantly higher than that in the CG group during the late stage of carcass decomposition, while the pH was significantly lower than that in the CG group. The NO_3_-N, NH_4_-N, and TN levels in the EG group also gradually decreased. We suggest that changes in community similarity between CG and EG were linked with the changes in physiochemical properties. For example, Hartman et al. and Lauber et al. reported that soil pH strongly influences the microbial diversity. The decrease in soil pH led to a decrease in microbial diversity [73,74]. Moreover, the decay of corpses was found to elevate the levels of NO_3_-N and NH_4_-N in the surrounding soils, leading to soil acidification and indirectly inhibiting the viability and activity of microbial communities in the soils, particularly phosphorus-solubilizing microbes [72]. These results support the hypothesis that cadaver decomposition simplifies the diversity of *phoC* and *phoD* harboring microbes. These findings suggest that animal carcass decomposition may compromise the efficiency and functional stability of resource acquisition and nutrient cycling within ecosystems [75]. This reveals that animal carcasses serve not only as a nutrient source but also as a biotic disturbance capable of homogenizing key soil microbial functions, warranting attention in the management and conservation of fragile ecosystems.

In addition to the differences in community structure, the temporal succession pattern of microbial community is also worth discussing. In agreement with Liang et al., we suggest that TDR reflects that the temporal turnover rate of microorganisms mainly depends on the environmental changes in a short time [76]. In this study, the steeper turnover rate showed that the cover speed of microbial communities carrying *phoC* and *phoD* functions in the corpse group within 94 days was significantly higher than that in the control group. The possible explanation is that the corpse, as an environmental disturbance, changed the surrounding physiochemical parameters (NO_3_-N, NH_4_-N, and TP increased and pH decreased), which may promoted the elimination and substitution of microbial species and reduced the diversity of microorganisms [77,78]. With the change in time turnover rate, the composition and structure of microbial communities will be adjusted [79]. For example, the significant decrease in *phoC* carrying microbes *Bosea*, and the decrease in microbes *Roseivivax and Variibacter* carrying *phoD*, while the increase in *Shinella* to cope with environmental changes and further adjust the time dynamics (Figure S5). In view of the fact that the change in microbial community composition is often related to the change in functional ability, it is necessary to further study the temporal succession mode of phosphate-solubilizing microbial community during the decomposition of corpses and its ecological function process.

### 4.3. The Plateau Pikas Corpse Decomposition Process of Corpse Leads to the Difference in the Assembly Process of phoC and phoD Microbial Communities in Soils

The microbial community assembly process has been considered as an important driving factor of microbial community structure and function in succession process [80]. Numerous studies have demonstrated that the assembly of microbial communities in natural environments is primarily driven by stochastic processes, as evidenced in various settings such as microbial electrolytic cell reactors [81], microbial community in Shackleton glacier, Antarctica [82], sustainable intensive agricultural soils [83], and microorganisms in tidal zone of estuary [84]. Our study revealed that the stochastic process predominantly governed the community assembly of phosphate-solubilizing microbes in the soils of the control group. However, as the decomposition and succession of corpses progressed, the assembly of microbial communities carrying the *phoC* gene exhibited a deterministic process, while the assembly of microbial communities with *phoD* during corpse decomposition continued to be governed by stochastic processes. The results of the combined PLS-PM combined with random forest showed that pH significantly affected the construction process of *phoC* microbial communities, but not *phoD* microbes (Figure 4c,d and Figure 7). One possible explanation may be the decrease in pH during corpse decomposition represents an environmental pressure that influences the adaptive environmental selection of populations in a specific habitat, leading to a deterministic assembly process for *phoC* microbial communities [85]. Previous research has also highlighted that in acidic soils, characterized by low pH and nutrient limitations, the soil environment favors the presence of *phoC* microbes, which play a crucial role in enhancing soil phosphorus availability, increasing acid phosphatase (ACP) activity, and improving phosphorus solubilization capacity [86]. Another explanation is that the *phoD* family is widely distributed among soil microbes and is particularly adapted to environments with varying phosphorus availability. These genes are often upregulated during phosphate starvation, indicating their role in phosphorus acquisition under nutrient-limited conditions [87,88]. While the *phoC* gene is less commonly discussed in the literature compared to *phoD*. It is typically found in specific microbial niches and may be involved in more specialized phosphorus cycling processes [88].

### 4.4. Plateau Pikas Corpse Decay May Increase the Complexity of phoC Microbial Network, but May Decrease the Complexity of phoD Microbial Network

Microbial communities are known to adapt to environmental changes by forming interaction networks, highlighting the importance of comprehending their interaction modes [89]. In this study, the network structure of EG group of *phoC* microbes was more closely connected, and the complexity was higher than that of CG group. The topological characteristics also proved this point. The multifunctionality of the microbiota in the ecosystem is positively related to the complexity of the whole community [90]. Therefore, the increased complexity of *phoC* but not *phoD* microbial community in the cadaver group in this study suggested cadaver decomposition may stimulate the synthesis of acid phosphatase (ACP) by *phoC*-bearing microbes to facilitate the breakdown of organophosphorus compounds [86]. This phenomenon can be explained by the decrease in pH caused by cadaver decomposition. The number of edges, positive edge rate, average degree, and graph density of microbial network in *phoC* EG group were significantly positively correlated with pH, while these topological features of *phoD* microbial network were significantly negatively correlated with pH (Figure 6c,d). This suggests that the low-pH environment was more conducive to the growth of the *phoC* microbial community compared to the *phoD* microbial community, thus fostering enhanced cooperation among networks [74,91]. In addition, the increase in both *phoC* and *phoD* modularity means that the decomposition of corpses enhanced the functional redundancy of these microbial communities [92]. Although the composition of the community changed, groups with the same function were able to maintain their functions effectively, ensuring the stability of community functions even after disturbances [93]. This correlation of species co-occurrence or mutual exclusion patterns observed in this study cannot prove the existence of direct ecological interaction between species [94]. In the future, various methods must be integrated to definitively verify and clarify the mechanism of microbial interaction.

## 5. Conclusions

In this study, the influence of the decomposition of animal carcasses on the community dynamics of phosphorus-solubilizing microbes carrying *phoC* and *phoD* in soils was explored. The decomposition of cadavers reduced the diversity of soil phosphate-solubilizing microbial communities by changing soil properties. The time turnover rate of *phoC*- and *phoD*-PCMs in the corpse group was higher than that of in control group. The decomposition of the corpse reduced the diversity of soil phosphate-solubilizing microbial communities by changing soil properties. In addition, the decomposition of the corpse formed different community assembly process and co-occurrence network between *phoC*- and *phoD*-PCMs. This differentiation may be caused by the different adaptability of *phoC* and *phoD* microbial communities to the environment of physical and chemical properties changes caused by corpse decomposition. These findings provide insights into the ecological mechanism of phosphorus-solubilizing bacteria community changes during the degradation of corpses. They also provide a basis for management strategies that incorporate cadavers as ecological disturbance factors within fragile grassland ecosystems. The limitation of this study lies in the fact that it only investigated the changes in microscopic phosphorus-increasing microorganisms caused by carcass decomposition, and it was a small-sample experiment. Moreover, the indicators of local temperature, humidity, and more phosphorus fractions were not detected. Future experiments can explore the effects of animal carcass decomposition on phosphorus fractions, the activity of phosphorus-increasing microorganisms, and the activities of ACP and ALP enzymes in the Qinghai–Tibet Plateau on a large sample size.

## Data Availability

The raw metagenomic data were available by accession number PRJEB75176 (http://www.ebi.ac.uk/ena/data/view/PRJEB75176) (accessed on 19 April 2025).

## Supplementary Materials

The following supporting information can be downloaded at: https://www.mdpi.com/article/10.3390/microorganisms14010153/s1, Figure S1: Rarefaction curve shows the sequencing depth of phoC and phoD, and the curve tends to be flat, indicating that the sequencing data of the sample is reasonable; Figure S2: The line chart shows the difference of soil physical and chemical factors between the experimental group and the control group, and the significance of the difference is tested by Kruskal-Wallis; Figure S3: Venn diagram shows that phoC and phoD phosphate-solubilizing microbes EG group and CG share OTUs; Figure S4: Box diagram shows the difference of alpha diversity between EG group and CG group of phoC and phoD microbial communities, and the PCoA diagram shows the difference of beta diversity between EG group and CG group of phoC and phoD microbial communities; Figure S5: Fit of the neutral community model (NCM) of microbial community assembly. The predicted occurrence frequencies for phoC microbes and phoD microbes; Figure S6: The significant fitting in relative abundance of phoC and phoD microbes of experimental group during corpse decay; Table S1: Kruskal-Wallis test shows the effects of corpse decay and time on soil physical and chemical factors; Table S2: Two-way ANOVA analysis shows the influence corpse decay and time on soil physical and chemical factors; Table S3: Physicochemical properties of soil samples in this study; Supplemental methods: PCR reaction system of phoC and phoD genes.
