# Supplementary material for: Divergent Successional Patterns of phoC- and phoD-Phosphate-Solubilizing Microbes During Plateau Mammal (Ochotona curzoniae) Carcass Decomposition
Source: Microorganisms. 2026 Jan 9;14(1):153. doi: 10.3390/microorganisms14010153 (PMC12843751; doi:10.3390/microorganisms14010153)
Supplement: Supplementary file 1 [file microorganisms-14-00153-s001.zip › microorganisms-4027531-supplementary.pdf]

Divergent successional patterns of *phoC*- and *phoD*- phosphate-solubilizing microbes during plateau mammal (*Ochotona curzoniae*) carcass decomposition

Jie Bi[a#], Xianxian Mu[a#], Shunqin Shi[a], Xueqian Hu[a], Petr Heděnec[c], Maoping Li[d*] and Huan Li[a, b*]

a. School of Public Health, Lanzhou University, Lanzhou 730000, China.

b. Center for Grassland Microbiome, Lanzhou University, Lanzhou, 730000, China.

c. Institute for Tropical Biodiversity and Sustainable Development, University Malaysia Terengganu, 21030, Kuala Nerus, Terengganu, Malaysia.

d. State Key Laboratory of Herbage Improvement and Grassland Agro-Ecosystems, and College of Pastoral Agriculture Science and Technology, Lanzhou University, Lanzhou, Gansu 730000, China.

[#]These authors contribute to this paper equally.

*Corresponding authors, *E-mail: lihuanzky@163.com (H.L.); Tel. +86-0931-8915008; Fax +86-0931-8915008.

Running title: Gravesoil *phoC*- and *phoD*- phosphate-solubilizing microbes

Supporting files:

Supplemental methods

Fig. S1-S6.

Table S1-S3.

Supplemental methods

1.1 PCR reaction system of phoC and phoD genes

The two-step protocol for *phoC* was as follows: 3 min at 95 °C, followed by 45 cycles of 10 s melt at 95 °C and 30 s anneal and elongation at 58 °C. The two-step protocol for *phoD* was as follows: 4 min at 94 °C, followed by 40 cycles of 30 s melt at 94 °C and 30 s anneal and elongation. A melt step followed the amplification to ensure specificity of the reaction, from 65 °C to 95 °C ramping 0.5 °C every 5 s [43].

43. Fraser, T.D.; Lynch, D.H.; Gaiero, J.; Khosla, K.; Dunfield, K.E. Quantification of bacterial non-specific acid (*phoC*) and alkaline (*phoD*) phosphatase genes in bulk and rhizosphere soil from organically managed soybean fields. *Appl. Soil Ecol.* **2017**, 111, 48–56.

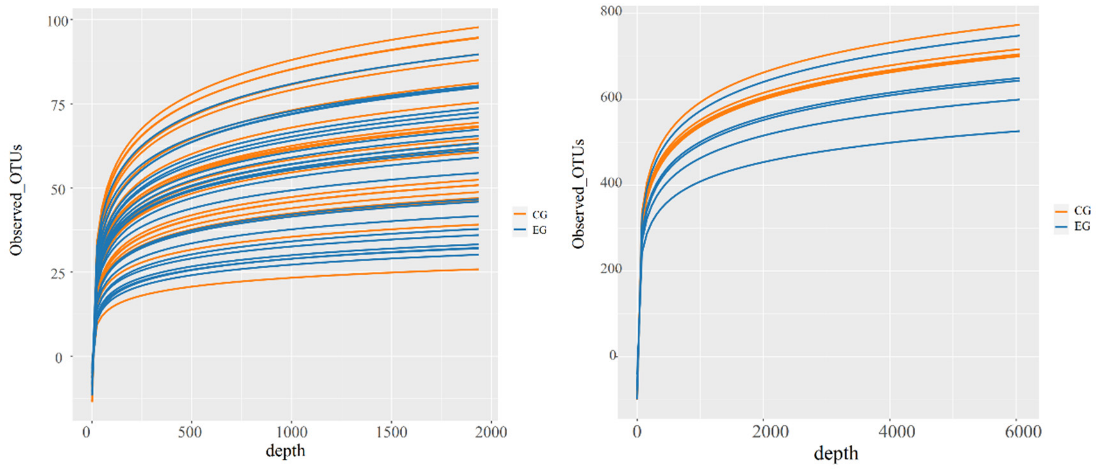

Fig. S1 Rarefaction curve shows the sequencing depth of *phoC* and *phoD*, and the curve tends to be flat, indicating that the sequencing data of the sample is reasonable.

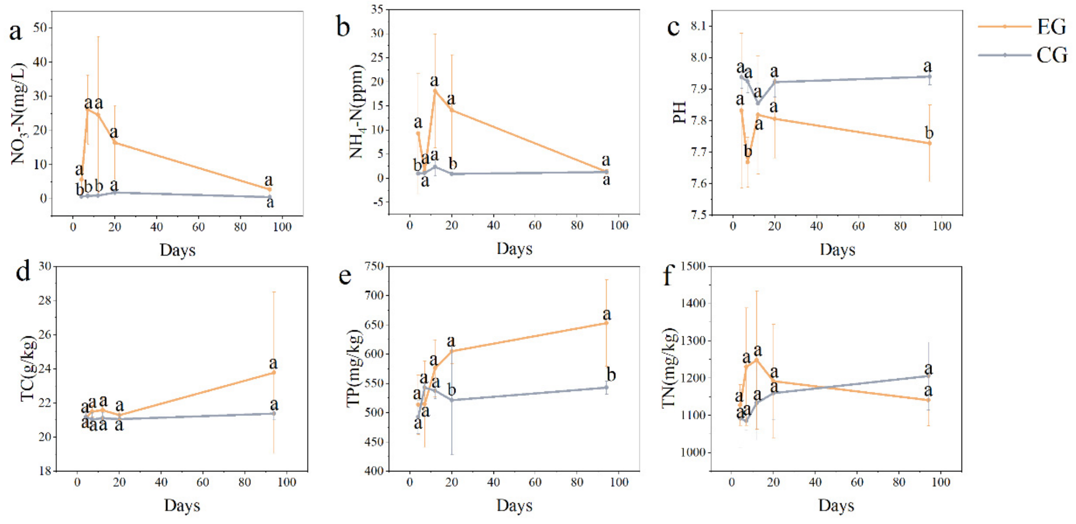

Fig. S2 The line chart shows the difference of soil physical and chemical factors between the experimental group and the control group, and the significance of the difference is tested by Kruskal-Wallis.

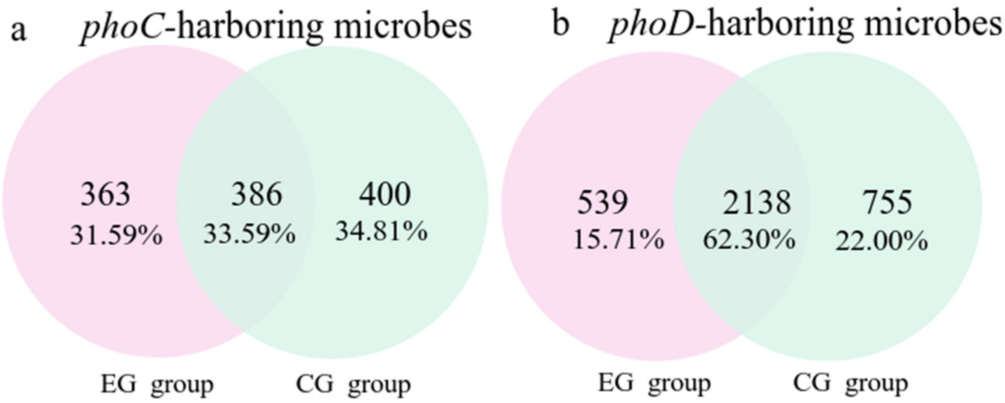

Fig. S3 Venn diagram shows that *phoC* and *phoD* phosphate-solubilizing microbes EG group and CG share OTUs.

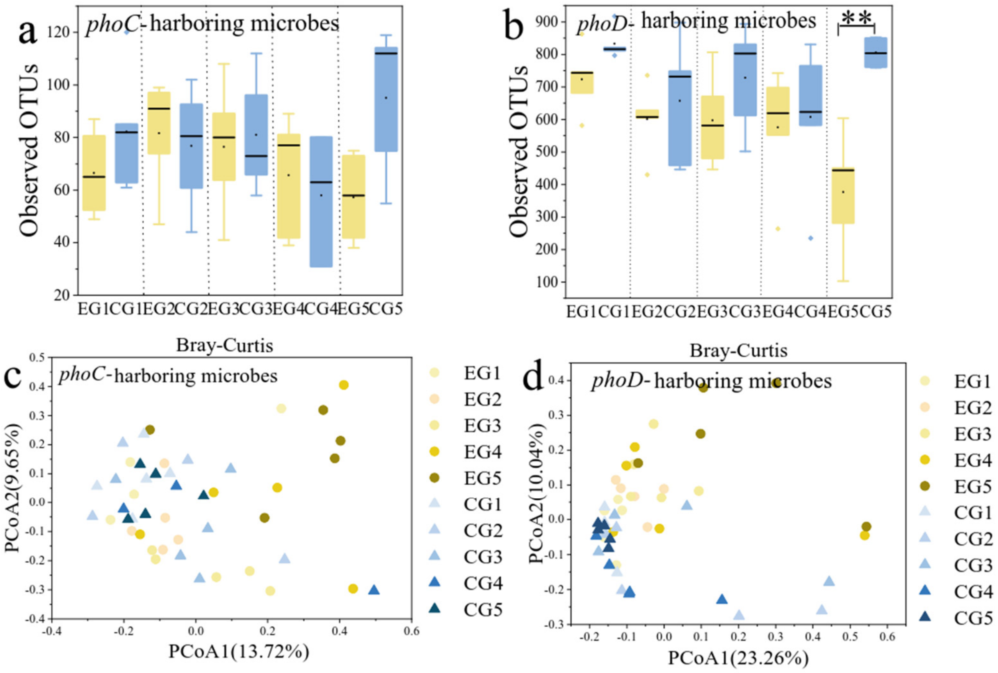

Fig. S4 Box diagram shows the difference of alpha diversity between EG group and CG group of *phoC* and *phoD* microbial communities, and the PCoA diagram shows the difference of beta diversity between EG group and CG group of *phoC* and *phoD* microbial communities. Abbreviations: EG, experimental group; CG, control group; EG1, EG2, EG3, EG4, EG5 experimental group at 4th, 7th, 12th, 20th and 94th day of corpse decomposition; CG1, CG2, CG3, CG4, CG5, control group at 4th, 7th, 12th, 20th and 94th day of corpse decomposition.

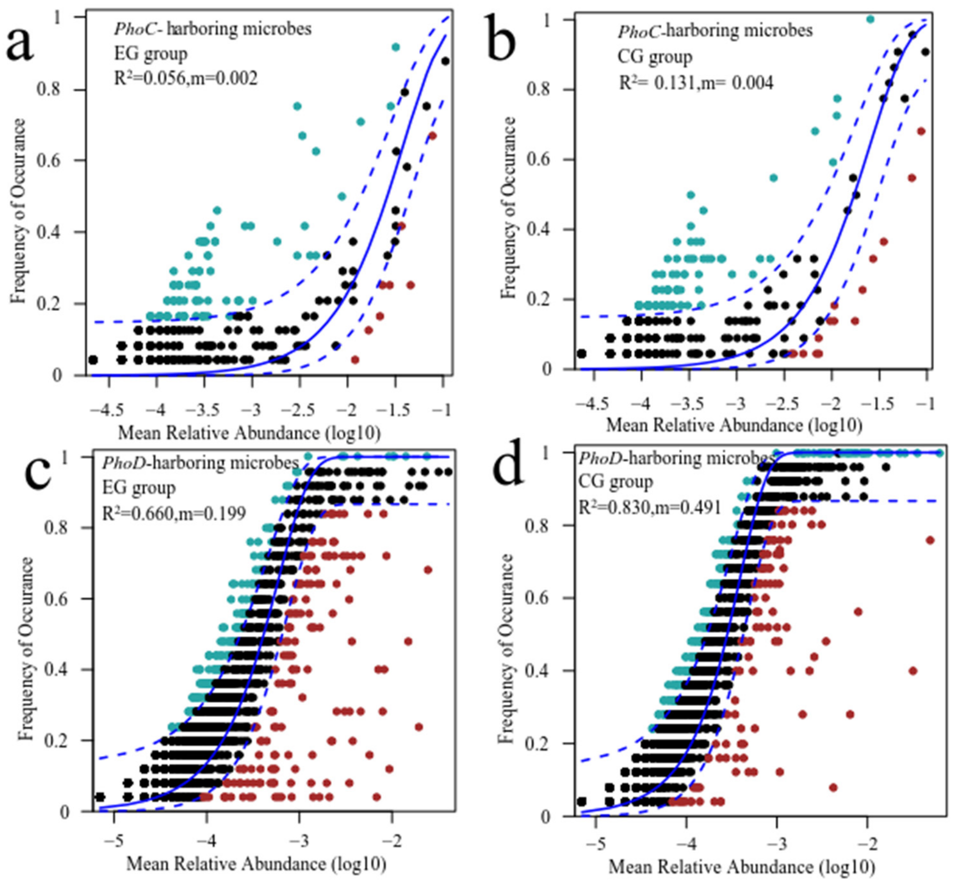

Fig. S5 Fit of the neutral community model (NCM) of microbial community assembly. The predicted occurrence frequencies for *phoC* microbes (a,b) and *phoD* microbes (d,e) Abbreviation: EG experimental group, CG control group.

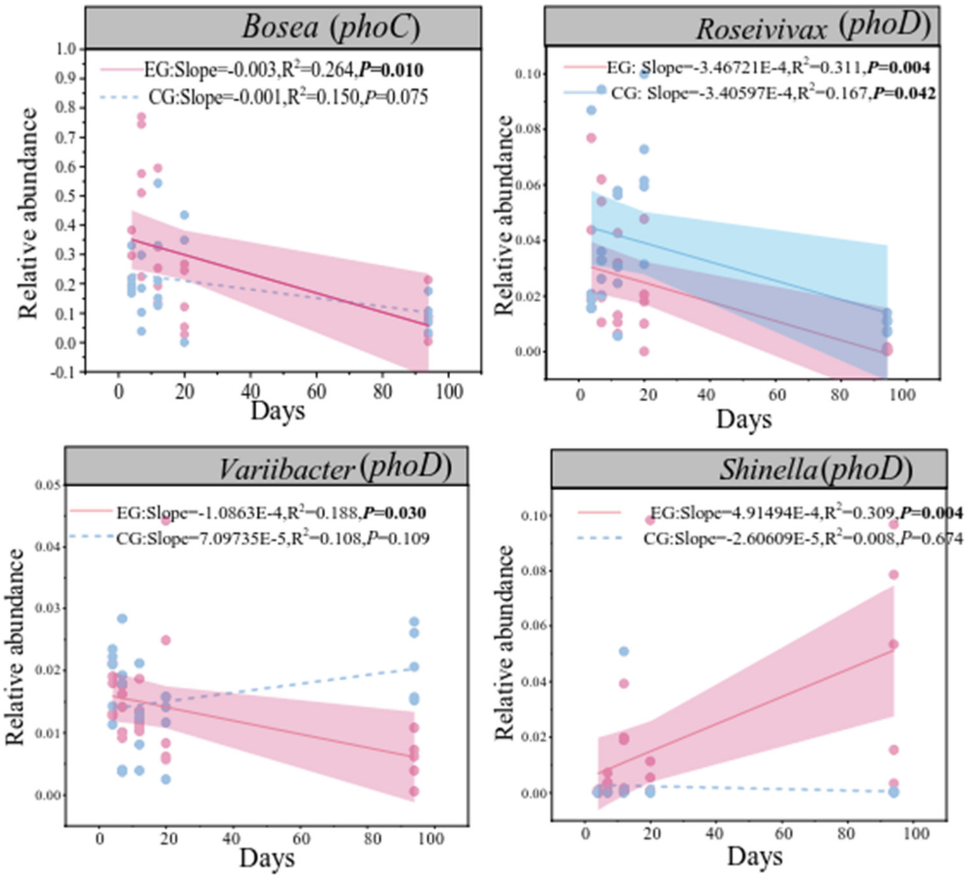

Fig. S6 The significant fitting in relative abundance of *phoC* and *phoD* microbes of experimental group during corpse decay.

Table S1 Kruskal-Wallis test (K-W test) shows the effects of corpse decay and time on soil physical and chemical factors.

Kruskal-Wallis test	χ^2	*P*
NO_3-N	41.582	**0.000**
NH_4-N	23.588	**0.005**
pH	33.659	**0.000**
TC	12.937	0.165
TP	26.4	**0.002**
TN	8.755	0.460

Table S2. Two-way ANOVA analysis (T-W ANOVA) shows the influence corpse decay and time on soil physical and chemical factors.

Two-way ANOVA	Treatment		Time		Treatment*Time	
	F	*P*	F	*P*	F	*P*
NO_3-Nmg/L	32.64 7	**0.000**	3.884	**0.009**	3.600	**0.013**
NH_4-Nppm	16.48 1	**0.000**	3.560	**0.014**	3.023	**0.029**
PH	18.53 2	**0.000**	0.799	0.533	1.365	0.263
TC(mg/kg)	2.592	0.115	1.510	0.218	0.977	0.431
TP(mg/kg)	9.670	**0.003**	4.947	**0.002**	2.816	**0.038**
TN(mg/kg)	2.905	0.096	0.821	0.520	1.411	0.248

Table S3. Physicochemical properties of soil samples in this study.

Group	Days	NO_3-N (mg/L)	NH_4-N (ppm)	TC (g/kg)	TP (mg/kg)	TN (mg/kg)	pH
EG1	4	1.760	30.969	20.944	486.803	1195.588	8.260
EG1	4	12.830	1.647	21.490	440.401	1108.969	7.660
EG1	4	6.030	3.526	21.556	568.446	1173.626	7.690
EG1	4	3.690	1.361	20.291	549.970	1069.683	7.740
EG1	4	3.820	9.098	21.465	521.884	1089.892	7.810
EG2	7	35.270	1.316	21.536	561.544	1480.694	7.570
EG2	7	37.130	1.363	21.546	399.802	1249.012	7.640
EG2	7	13.690	1.089	21.217	490.142	1066.563	7.710
EG2	7	25.560	2.110	22.032	534.820	1221.733	7.640
EG2	7	18.880	1.184	21.182	587.950	1134.745	7.780
EG3	12	5.940	29.325	20.913	534.147	1080.594	7.990
EG3	12	58.580	2.028	21.612	590.747	1071.669	7.570
EG3	12	4.660	29.687	21.212	596.824	1220.534	8.020
EG3	12	17.820	17.632	21.313	519.969	1375.172	7.730
EG3	12	36.210	11.884	22.827	637.744	1495.464	7.780
EG4	20	13.440	20.646	20.984	625.664	1213.274	7.880
EG4	20	8.780	3.736	21.004	569.726	945.145	7.800
EG4	20	35.370	1.323	21.460	606.766	1318.831	7.610
EG4	20	14.370	16.497	21.475	605.824	1169.102	7.800
EG4	20	10.280	28.466	21.541	618.431	1313.725	7.940
EG5	94	3.800	1.455	21.698	640.227	1165.394	7.710
EG5	94	3.250	2.067	22.042	594.249	1239.905	7.740
EG5	94	0.320	1.124	32.218	595.778	1087.568	7.540
EG5	94	5.310	1.029	21.450	774.014	1066.478	7.780
EG5	94	1.120	1.059	21.470	662.321	1145.462	7.870
CG1	4	0.760	0.731	21.217	524.356	984.158	7.900
CG1	4	0.680	0.919	20.918	508.582	1103.176	7.900
CG1	4	0.520	1.404	21.197	491.797	1075.114	7.950
CG1	4	0.780	1.001	21.845	489.715	1096.519	7.980
CG1	4	0.680	0.835	20.771	449.366	1199.267	7.960
CG2	7	0.610	1.131	21.014	536.621	1092.833	7.890
CG2	7	0.830	0.822	20.797	547.995	1099.743	7.920
CG2	7	1.010	1.293	20.478	540.715	1043.253	7.970
CG2	7	1.170	1.501	21.379	538.742	1100.256	7.890
CG2	7	0.900	0.808	21.490	551.513	1090.370	7.950
CG3	12	1.310	1.908	21.435	556.785	1189.117	7.800
CG3	12	0.950	5.711	21.212	545.765	1163.934	7.910
CG3	12	0.690	1.613	21.080	525.988	1019.269	7.820
CG3	12	0.820	1.087	21.293	523.847	1041.880	7.940
CG3	12	1.210	1.545	20.523	535.373	1252.387	7.800
CG4	20	2.240	0.978	21.009	394.954	1143.236	7.880
CG4	20	1.140	0.867	21.531	544.502	1169.897	7.870
CG4	20	1.620	0.868	20.944	525.741	1135.823	7.970
CG4	20	2.300	0.882	21.009	489.959	1078.559	7.970
CG4	20	1.950	0.887	20.797	653.406	1272.655	7.920
CG5	94	0.720	1.334	21.197	528.878	1134.930	7.950

CG5	94	0.420	1.279	21.956	546.143	1307.261	7.910
CG5	94	0.690	1.356	21.303	546.792	1238.768	7.930
CG5	94	0.570	1.484	21.455	557.745	1088.195	7.980
CG5	94	0.330	0.914	20.974	534.419	1259.855	7.930
